# Cryo-EM structures of human bradykinin receptor-G_q_ proteins complexes

**DOI:** 10.1038/s41467-022-28399-1

**Published:** 2022-02-07

**Authors:** Jinkang Shen, Dongqi Zhang, Yao Fu, Anqi Chen, Xiaoli Yang, Haitao Zhang

**Affiliations:** 1 Hangzhou Institute of Innovative Medicine, Institute of Pharmacology and Toxicology, Zhejiang Province Key Laboratory of Anti-Cancer Drug Research, College of Pharmaceutical Sciences, Zhejiang University, Hangzhou, Zhejiang 310058 China; 2 The Second Affiliated Hospital, Zhejiang University School of Medicine, Hangzhou, Zhejiang 310009 China

**Keywords:** Receptor pharmacology, Cryoelectron microscopy, G protein-coupled receptors

## Abstract

The type 2 bradykinin receptor (B2R) is a G protein-coupled receptor (GPCR) in the cardiovascular system, and the dysfunction of B2R leads to inflammation, hereditary angioedema, and pain. Bradykinin and kallidin are both endogenous peptide agonists of B2R, acting as vasodilators to protect the cardiovascular system. Here we determine two cryo-electron microscopy (cryo-EM) structures of human B2R-G_q_ in complex with bradykinin and kallidin at 3.0 Å and 2.9 Å resolution, respectively. The ligand-binding pocket accommodates S-shaped peptides, with aspartic acids and glutamates as an anion trap. The phenylalanines at the tail of the peptides induce significant conformational changes in the toggle switch W283^6.48^, the conserved PIF, DRY, and NPxxY motifs, for the B2R activation. This further induces the extensive interactions of the intracellular loops ICL2/3 and helix 8 with G_q_ proteins. Our structures elucidate the molecular mechanisms for the ligand binding, receptor activation, and G_q_ proteins coupling of B2R.

## Abstract (web summary)

Type 2 bradykinin receptor (B2R) is essential in vasodilation and cardioprotection. Here the authors present two cryo-EM structures of human B2R-Gq in complex with bradykinin and kallidin to elucidate the mechanisms for ligand binding, receptor activation, and Gq proteins coupling.

## Introduction

The kallikrein-kinin system (KKS) consists of kininogens, kallikrein enzymes, kinins, type 1 and type 2 bradykinin receptors (B1R and B2R), which plays important roles in vasodilation, inflammation, vascular permeability, cardioprotection, coagulation, and pain^1–4^. Kinins are short-lived peptides of various lengths and they are produced by enzymatic hydrolysis of precursor kininogens^5^. One derivative from the high molecular weight kininogen is called bradykinin (RPPGFSPFR), and another derivative from the low molecular weight kininogen is called kallidin (KRPPGFSPFR)^6–8^. Both kinins with only one N-terminal lysine difference are essential bioactive factors that regulate multiple physiological and pathological progress including blood circulation, smooth muscle contraction, and inflammatory responses. Increasing levels of kinins or dysfunction of their receptors lead to hereditary angioedema, an autosomal dominant disorder that is characterized by swelling of mucosal, submucosal tissue or subcutaneous^9,10^. The coronavirus disease (COVID-19) patients got pulmonary edema in the early stage mainly due to the increasing levels of B1R and B2R activation as well as their agonists stimulation^11–14^. And the B2R selective antagonist icatibant (D-Arg-Arg-Pro-Hyp-Gly-Thi-Ser-D-Tic-Oic-Arg) was suggested to relieve the symptoms^15^.

B1R and B2R belong to the γ-branch of class A G protein-coupled receptors (GPCRs) sharing the sequence identity of 32%. B2R is constitutively expressed in human tissues^8^. While, B1R is expressed robustly in the conditions of inflammation and oxidative stress^16^. Both B1R and B2R signal through G_q_ proteins pathway which promotes the phospholipase C activation and calcium mobilization^17,18^. Both the peptide ligands bradykinin and kallidin are endogenous full agonists of the human B2R^19^. The binding affinity of bradykinin is much higher for B2R than B1R. While, the endogenous B1R peptide agonist desArg^10^-kallidin (KRPPGFSPF) lacking the C-terminal arginine shows 100,000-fold higher affinity for B1R than B2R^20,21^.

Extensive efforts on developing new antagonists with higher potency and selectivity were hampered by the lacking of three-dimensional structures of B2R, and the molecular mechanisms for the ligand binding, receptor activation, and G_q_ proteins coupling were still elusive.

Here we determine two cryo-electron microscopy (cryo-EM) structures of B2R-G_q_ complexes in the presence of bradykinin and kallidin, respectively. Our structures reveal the critical interactions in the ligand-binding pocket and on the B2R-G_q_ interface, which shed light on a better understanding of the ligand selectivity and G proteins selectivity.

## Results

### Cryo-EM structure determination of B2R-G_q_ complexes

**Fig. 1:**
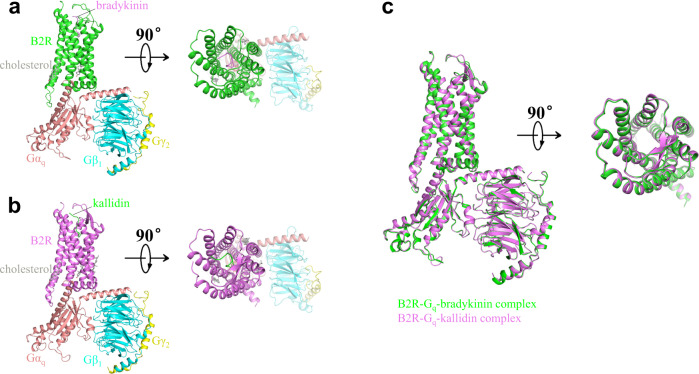
Overall architectures of B2R-G_q_ complexes. **a**, **b** Models for the B2R-G_q_ in complex with bradykinin (**a**, B2R, green; bradykinin, violet) and kallidin (**b**, B2R, violet; kallidin, green). **c** Structural comparison of bradykinin-bound (green) and kallidin-bound (violet) B2R.

To obtain the active structures of B2R in complex with G_q_ heterotrimer, two B2R full agonists bradykinin and kallidin were used for the complex formation. The human B2R protein was engineered by replacing the N-terminal 28 amino acids with a maltose-binding protein (MBP) without any mutations (Supplementary Fig. 1). The engineered B2R used for the structure determination showed identical downstream calcium signaling to the wild-type (WT) B2R, indicating the N-terminal truncation and MBP fusion did not alter the B2R function (Supplementary Fig. 2a, Supplementary Table 1). The human Gα_q_ protein was modified with two dominant-negative mutations R183Q and Q209L to stabilize the complexes^22,23^. The ability of the double-mutated G_q_ to activate the downstream calcium signaling was significantly decreased compared to the WT-G_q_, since the R183Q mutation around the nucleotide-binding site decreased the affinity of GDP by disrupting the hydrogen bonds between R183 and GDP molecule^23^ (Supplementary Fig. 2a, Supplementary Table 1). The human Gβ_1_ and Gγ_2_ proteins were both wild types. GPCR-G proteins complexes were usually determined using antibodies or NanoBit tethering technology to stabilize the complexes^24–26^. However, our B2R-G_q_ complexes were found to be very stable during the purification and cryo-EM procedures without these stabilizing agents, and thus our B2R-G_q_ structures might represent more natural conformations. The B2R-G_q_ proteins were co-expressed in the insect cells and solubilized in lauryl maltose neopentyl glycol (LMNG) with agonists. During the cryo-EM data processing, 2D classification revealed averages with clear α-helices for the complex and 3D classification identified the desired particle partition showing well-defined features for the complex (Supplementary Figs. 3 and 4). The two structures of B2R-G_q_ in complex with bradykinin and kallidin were determined at the resolutions of 3.0 and 2.9 angstrom, respectively (Fig. 1; Supplementary Table 2). The overall structure of bradykinin-bound B2R was almost identical to that of kallidin-bound B2R, with Cα root mean square deviation (R.M.S.D.) values of 0.240 Å for the whole complex, 0.259 Å for B2R, 0.211 Å for Gα_q_, 0.214 Å for Gβ_1_, and 0.351 Å for Gγ_2_ (Fig. 1). We mainly focused on the B2R-G_q_-kallidin complex for the following discussion unless otherwise indicated. From the cryo-EM density maps, the residues of transmembrane helices (TMs) of B2R could be clearly identified (Supplementary Fig. 5). However, the N-terminus of B2R (Leu29 to Lys46) and the α-helical domain (AHD) of G_q_ showed weak densities due to flexibility in these regions like many other GPCR-G protein complex structures. The densities of the orthosteric ligand-binding pocket and B2R-G_q_ interface were unambiguous, enabling near-atomic modeling building for the ligand binding and G protein coupling (Supplementary Fig. 5).

### Overall architectures of B2R-G_q_ complexes

B2R shares the sequence identity of 28% and 27% to the angiotensin receptors AT_1_R and AT_2_R, respectively, which are also GPCRs regulating the cardiovascular system and binding the endogenous peptide agonist angiotensin II (DRVYIHPF). Superposition of the G_q_-bound active B2R structure with the inactive AT_1_R structure^27^ revealed a 9.0 Å outward movement of the transmembrane helix 6 (TM6) when measuring the Cα atoms of the residues at 6.31 (Ballesteros-Weinstein numbering)^28^, as shown in Supplementary Fig. 6b. Besides, at the cytoplasmic side, there was a 2.4 Å outward shift of TM1 and a 2.7 Å inward shift of TM7. Compared to the active AT_1_R structure^29^, a 6.3 Å inward movement of TM5 in B2R induced substantial compaction of the G protein binding pocket for signal transduction (Supplementary Fig. 6a). However, the transmembrane helices of both antagonist-bound^30^ and agonist-bound^31^ AT_2_R showed active conformations, similar to the active B2R structure (Supplementary Fig. 6d). The helices 8 of the active AT_1_R^29^ and other GPCR-G protein complexes, such as β_2_ adrenergic receptor (β_2_AR)-G_s_^32^, M1 muscarinic acetylcholine receptor (M1R)-G_11_^33^, and histamine H_1_ receptor (H_1_R)-G_q_^24^ lay proximately to the membrane, while the helix 8 of B2R adopted a ~25° downward rotation that was stabilized by the electrostatic interactions between R338^8.49^ of B2R and E355^G.H5.22^ of α5-helix of G_q_ (Supplementary Figs. 6a and 7). On the contrary, the helix 8 of antagonist-bound AT_2_R interacted with the intracellular tips of TM3, TM5, and TM6, functioning as a gatekeeper blocking the binding of G proteins (Supplementary Fig. 6d). At the extracellular side of B2R, except for the conserved disulfide bonds between C130^3.25^ and C211^ECL2^ bridging TM3 and extracellular loop 2 (ECL2), an additional disulfide bond linking the N-terminus and TM7 were observed between C47^N-term^ and C304^7.25^. Similar disulfide bonds were also found in AT_1_R^29^ and AT_2_R^31^, while in other GPCRs they might bridge the residues between ECL1 and ECL2^34^ or within ECL2^32^ or ECL3^24,35^ (Supplementary Fig. 8a). ECL2 of B2R formed a β-sheet which was a common feature in many peptide GPCRs such as AT_1_R, AT_2_R, and neurotensin receptor 1 (NTSR1)^29,30,36^. Interestingly, ECL2 of B2R on top of the ligand-binding pocket was stabilized by the hydrogen bonds with the residues D122^ECL1^, W123^ECL1^, and E127^3.22^ (Supplementary Fig. 8b). In the non-peptide GPCRs, ECL2 could form a short α-helix^32^ or interact with ECL1^35^. As shown in Supplementary Fig. 9, three structured steroids (cholesterols) were found in the intracellular clefts between TM2-TM4, TM3-TM4, and TM6-TM7, with extensive hydrophobic interactions, which might help maintain the functional conformations of B2R and regulate the movements of transmembrane helices.

### Molecular determinants of B2R-ligand binding

**Fig. 2:**
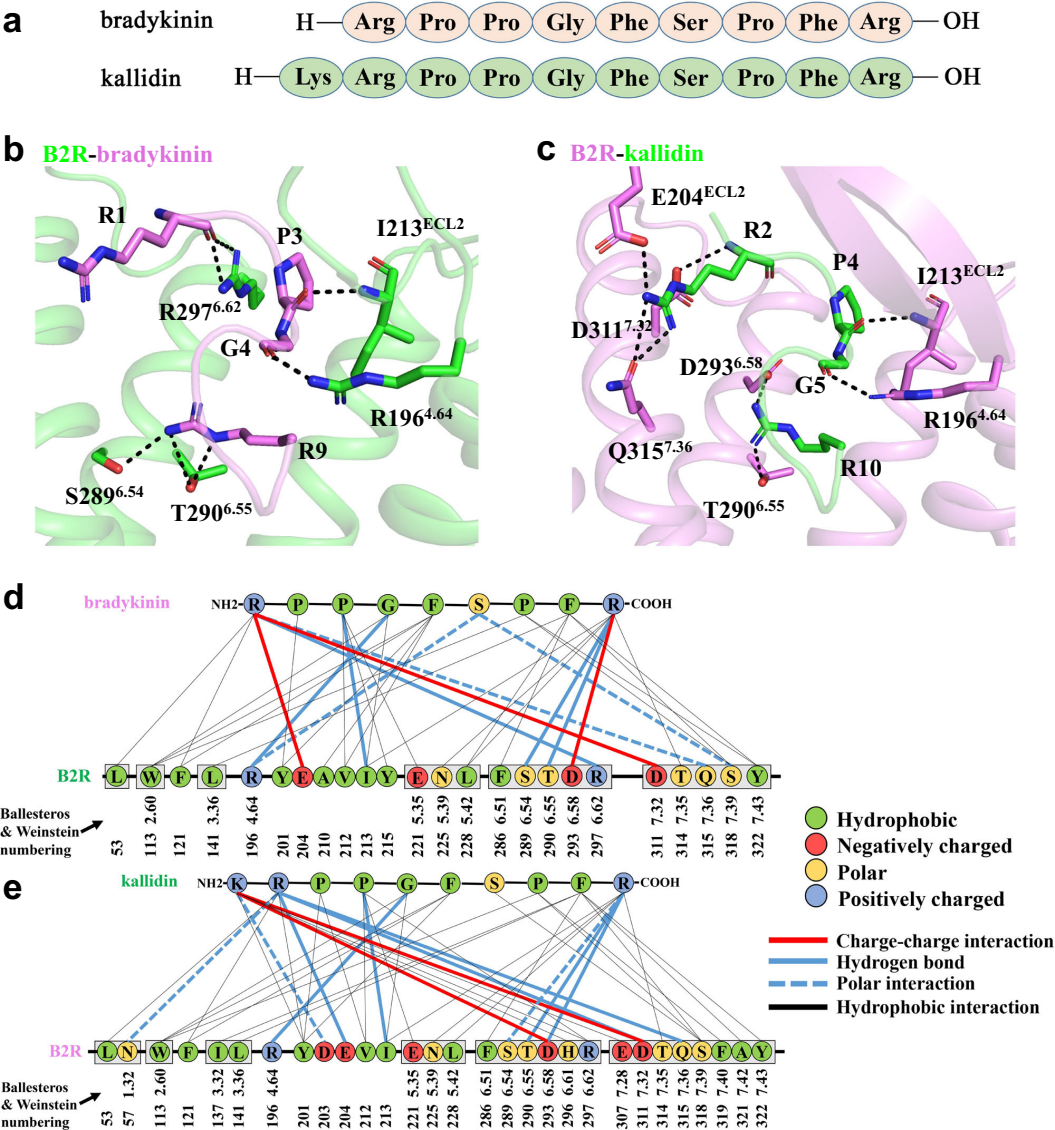
Molecular determinants of B2R-ligand binding. **a** Sequences of bradykinin and kallidin. **b**, **c** B2R-ligand interactions in the orthosteric ligand-binding pockets of the active B2R-G_q_ complexes with bradykinin (**b**, shown as violet sticks) and kallidin (**c**, shown as green sticks). Hydrogen bonds between B2R and agonists were labelled as dotted lines, and all residues participating in the interactions were shown as sticks. **d**, **e** Diagrams of the contacts between B2R and bradykinin (**d**) or kallidin (**e**).

In our determined B2R-G_q_ structures, both ligands bradykinin and kallidin adopted S-shaped conformations with their C-termini inserting deeply into the orthosteric binding pocket and their N-termini extending to the extracellular side (Fig. 2). Extensive hydrogen bonds, polar, and hydrophobic interactions were found for the ligands binding to B2R (Fig. 2). The bradykinin and kallidin were both positively charged at the N and C-termini, where the arginines or lysine formed polar interactions with glutamates or aspartic acids of B2R. In the kallidin-bound structure, R2 and R10 of kallidin (referred to R2^K^ and R10^K^, respectively) contributed most hydrogen bonds and polar interactions with B2R including N57^1.32^, E204^ECL2^, D311^7.32^, and Q315^7.36^ for R2^K^ and S289^6.54^, T290^6.55^, and D293^6.58^ for R10^K^. Pairs of P4^K^-I213^ECL2^ and G5^K^-R196^4.64^ were suggested to function as anchor points to avoid the conformational perturbation of kallidin and enhance its binding affinity. This was supported by our calcium mobilization assays that R196^4.64^A mutation completely abolished the kallidin-induced B2R activation to the downstream calcium signaling, and I213^ECL2^A mutation significantly decreased the kallidin-induced B2R activation with the EC_50_ value of 150-fold higher than the WT-B2R (Supplementary Fig. 2c, Supplementary Table 3). Bradykinin and kallidin shared similar interactions with B2R, except for the R1 of bradykinin (referred to R1^B^), which was at the equivalent position as R2^K^, formed the hydrogen bond with R297^6.62^ rather than Q315^7.36^, as well as the additional polar interactions and ionic interactions formed by K1^K^ with D203^ECL2^, D293^6.58^, and D311^7.32^.

Intriguingly, the charge distribution of the ligand-binding pocket determined the sigmoidal binding poses of the two peptides. Negatively charged residues such as glutamates and aspartic acids (D203^ECL2^, E204^ECL2^, E221^5.35^, D293^6.58^, E307^7.28^, and D311^7.32^) located at the entrance, while the bottom of the pocket was found to be mostly hydrophobic (Supplementary Fig. 10). Consequently, the positively charged R10^K^/R9^B^ bent upwards to form electrostatic interactions with D293^6.58^, which was further stabilized by the interactions between P4^K^/P3^B^ or G5^K^/G4^B^ and B2R. Moreover, two intramolecular hydrogen bonds G4^B^-R9^B^/G5^K^-R10^K^ and S6^B^-R9^B^/S7^K^-R10^K^ maintained the S-shaped conformations of the peptides (Supplementary Fig. 11c). Surprisingly, the side chains of D203^ECL2^, E204^ECL2^, E221^5.35^, D293^6.58^, E307^7.28^, and D311^7.32^ covered the ligand-binding pocket by forming an anion trap preventing the ligand dissociation by the electrostatic interactions. Hydrophobic interactions and van der Waals forces also played important roles for the ligand binding to B2R, P3^K^/P2^B^ with Y201^ECL2^, and P4^K^/P3^B^ with V212^ECL2^/E221^5.35^, as well as extensive hydrophobic contacts among F6^K^/F5^B^ with the residues from TM2, ECL1, and ECL2, and F9^K^/F8^B^ with the residues from TM3, TM6, and TM7 (Fig. 2d, e, Supplementary Fig. 2b).

A recent study also reported the bradykinin-bound B2R structure, as well as the desArg^10^-kallidin-bound B1R structure, using the NanoBit strategy^37^. The two B2R structures exhibited identical conformations with R.M.S.D. values of 0.595 Å (for Cα atoms in receptors). Comparing the ligand-binding pockets of our determined bradykinin-bound B2R structure with the desArg^10^-kallidin-bound B1R structure revealed the molecular basis for kinin selectivity on B1R and B2R^37^. DesArg^10^-kallidin was a bradykinin derivative lacking the C-terminal arginine but with the N-terminal lysine. It was obvious that K118^3.33^ and R202^5.38^ of B1R could form electrostatic interactions with F9 of desArg^10^-kallidin (referred to F9^DK^), while the cognate residues in B2R were S138^3.33^ and T224^5.38^, which were unable to provide polar interactions to stabilize F8^B^ (Supplementary Fig. 12b). Moreover, R9^B^ could not fit into the narrow pocket consisting of R202^5.38^, Y266^6.51^, and E273^6.58^ of B1R, and there would be a severe steric hindrance between R9^B^ and R202^5.38^ of B1R. On the contrary, the side chains of their cognate residues in B2R, T224^5.38^, F286^6.51^, and D293^6.58^, were small enough to provide space for R9^B^ (Supplementary Fig. 12c). These differences provided the structural basis for the preference of bradykinin to B2R and desArg^10^-kallidin to B1R.

The structures of kallidin-bound B2R, AngII-bound AT_1_R, and AngII-bound AT_2_R were quite similar with R.M.S.D. values of 1.26 Å for B2R/AT_1_R and 1.07 Å for B2R/AT_2_R (for all Cα atoms in receptors)^38–40^. All the peptide agonists in these structures adopted their C-termini settling at the bottom of pockets and N-termini extending to the extracellular side. The AngII in AT_1_R exhibited anti-parallel β strands with the N-terminus and ECL2, compared to the unstructured N-termini of AngII in AT_2_R and kallidin in B2R (Supplementary Fig. 13b). Although these peptides showed diverse conformations at the N-termini, backbones of them converged in the middle and at the C-termini. The crooked backbone at F9^K^ resulted in upward positioning of R10^K^, while it was absent in AngII. Intramolecular hydrogen bonds among G5^K^, S7^K^, and R10^K^ in kallidin, as well as Y4 and F8 in AngII, stabilized the C-terminal shapes of peptides (Supplementary Figs. 11c and 13c). The pocket entrances of B2R, AT_1_R, and AT_2_R were similar, and the negatively charged D^6.58^ and D^7.32^ at the entrances were highly conserved, which interacted with the positively charged arginines of kallidin and AngII (Supplementary Fig. 11b and 13d). In the middle of kallidin, hydrophobic residues P4^K^ and G5^K^ formed hydrogen bonds with I213^ECL2^ and R196^4.64^. Similar interactions were also found in AngII-bound AT_1_R (Supplementary Fig. 11d). F9^K^ in B2R and F8 in AT_1_R and AT_2_R were settled at the bottom of pockets forming extensive van der Waals forces to further stabilize the binding of peptides. Similar peptide agonist-binding patterns in the orthosteric pockets of B2R, AT_1_R, and AT_2_R indicated that they might share common activation mechanisms to propagate the extracellular signals to the intracellular G protein coupling.

### Molecular mechanisms of B2R-G_q_ coupling

**Fig. 3:**
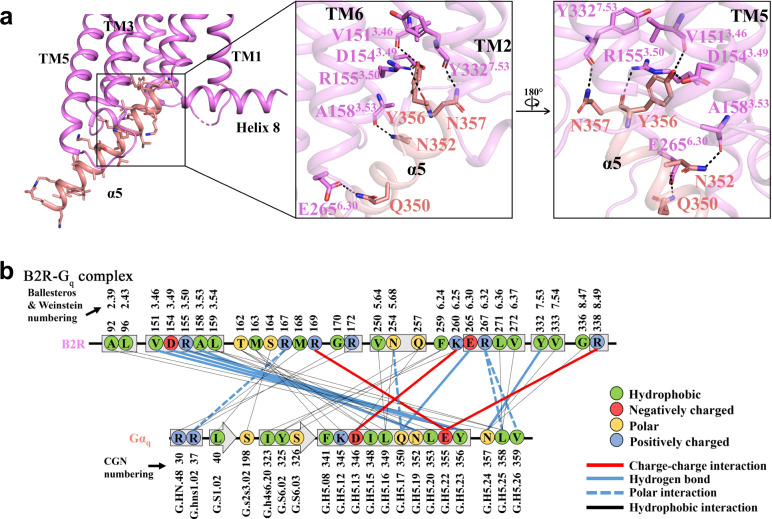
Molecular mechanisms of B2R-G_q_ coupling. **a** B2R-G_q_ interactions at the intracellular cleft of 7TMs and α5-helix of G_q_. B2R was colored in violet and G_q_ was colored in salmon. Residues with hydrogen bonds were shown as sticks and dotted lines. **b** Diagram of the B2R-G_q_ contacts.

Extensive interactions from TMs2-3, TMs5-7, ICLs2-3, and helix 8 of B2R, with the α5-helix, αN-helix, and αN-β1 loop of G_q_ on the B2R-G_q_ interface were found to stabilize the complex (Fig. 3). Y356^G.H5.23^ (CGN numbering^41^) at the tail of α5-helix formed multiple hydrogen bonds with V151^3.46^, and D154^3.49^-R155^3.50^ of the conserved DRY motif. The α5-helix of G_q_ also formed hydrogen bonds with A158^3.53^, E265^6.30^, and Y332^7.53^ of the conserved NPxxY motif. Several polar interactions were found between R167^ICL2^-R37^G.hns1.02^, N254^5.68^-Q350^G.H5.17^, and R267^6.32^-L358^G.H5.25^/V359^G.H5.26^. The helix 8 of B2R was also involved in the G_q_ coupling by the interactions between R338^8.49^-E355^G.H5.22^ and G336^8.47^-N357^G.H5.24^ (Fig. 3b).

**Fig. 4:**
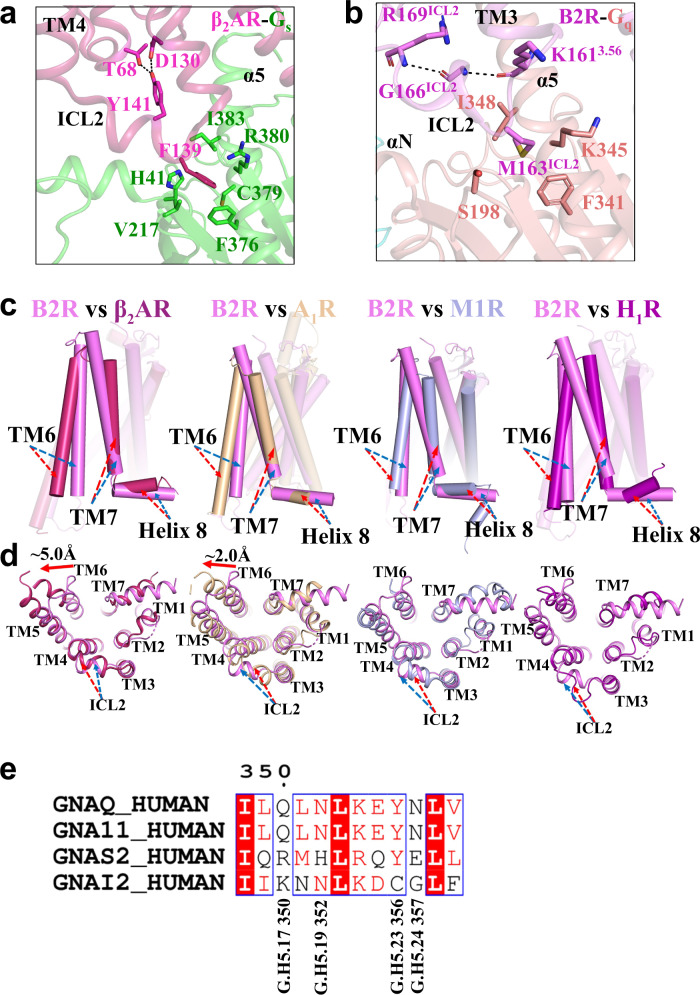
Comparison of various GPCR-G protein complexes. **a** In the β_2_AR-G_s_ complex, T68 and D130 formed hydrogen bonds with Y141 of ICL2 to stabilize the short α-helix. F139 of β_2_AR inserted into a hydrophobic pocket on the G_s_. **b** In the B2R-G_q_ complex, G166 formed hydrogen bonds with K161 and R169, and the α-helical conformation of ICL2 positioned M163 into a hydrophobic pocket on the surface of G_q_. The pocket was consisted of residues from α5-helix (F341, K345, I348) and β2-β3 loop (S198). **c**, **d** Lateral (**c**) and intracellular (**d**) views of the B2R-G_q_ (violet) superposed with the β_2_AR-G_s_ (warm-pink, PDB ID: 3SN6), A_1_R-G_i_ (wheat, PDB ID: 6D9H), M1R-G_11_ (light-blue, PDB ID: 6OIJ), and H_1_R-G_q_ (purple, PDB ID: 7DFL). **e** Sequence alignment of human G_q_, G_s2_, G_i2_, and G_11_ proteins.

The ICL2 of B2R adopted a short α-helix like that in the β_2_AR-G_s_ structure. Y141^ICL2^ of β_2_AR formed hydrogen bonds with T68^2.39^ and D130^3.49^ to stabilize the α-helical conformation of ICL2 (Fig. 4a)^32^. G166^ICL2^ of B2R formed two hydrogen bonds with R169^ICL2^ and K161^3.56^, while M163^ICL2^ interacted with F341^G.H5.08^, K345^G.H5.12^, and I348^G.H5.15^ in the α5-helix and S198^G.s2s3.02^ in the β2-β3 loop of G_q_ (Fig. 4b). In the β_2_AR-G_s_ structure, the intracellular tip of TM6 moved 14 Å outwards while only 9 Å in the B2R-G_q_ structures (Fig. 4c, d). It was suggested that the residues tyrosine and leucine at the C terminus of G_s_ required more space to accommodate the bulky side chains, compared to the smaller side chain of valine at the C terminus of G_q_. Additionally, a ~15° rotation of α5-helix towards TM1 was found in the B2R-G_q_ structure, compared to the β_2_AR-G_s_ structure (Supplementary Fig. 7).

Diverse interactions for G_q/11_-coupling were observed by comparing the B2R-G_q_ structures with the M1R-G_11_^33^ and H_1_R-G_q_ structures^24^. All α5-helices in these structures inserted into the intracellular cavities and tilted toward TM1 and TM7 (Supplementary Fig. 7). However, some additional contacts at the G_q_ and helix 8 interface were found in the B2R-G_q_ structures (Fig. 3b). The Q^G.H5.17^ and N^G.H5.24^ were highly conserved in the G_q/11_ family proteins and critical for the polar interactions with B2R, which might determine the G_q_ coupling of B2R. However, it was suggested that B2R could also couple to G_i_^42–44^, in which the common N^G.H5.19^ in the G_i2/11/q_ might form hydrogen bonds with B2R (Fig. 4e).

### Molecular insights of B2R activation

**Fig. 5:**
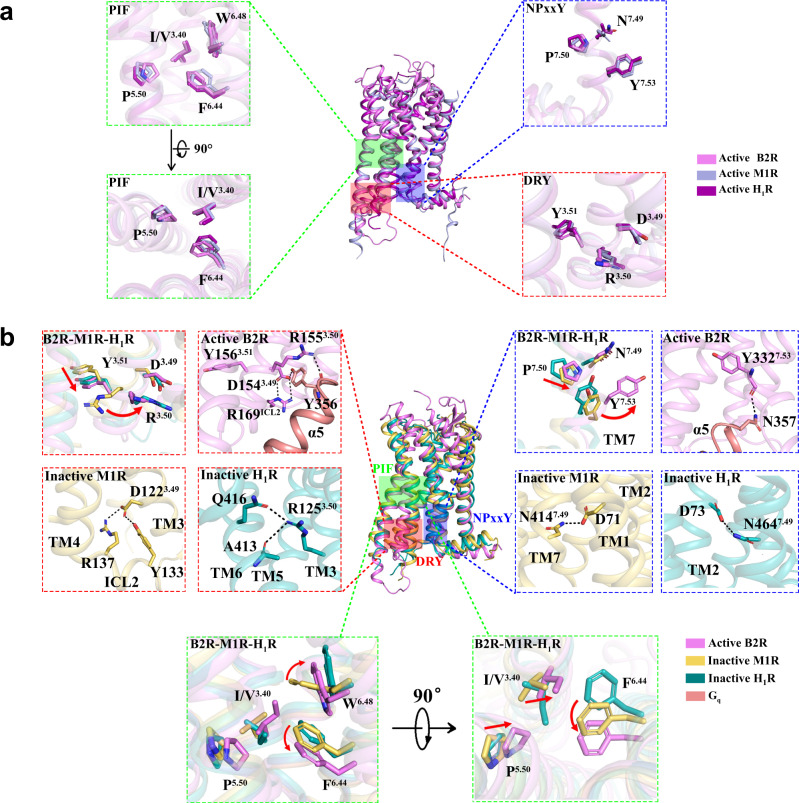
Molecular insights of B2R activation. **a** Structural comparison of active B2R (violet) with active M1R (light-blue, PDB ID: 6OIJ) and active H_1_R (purple, PDB ID: 7DFL). DRY motif, NPxxY motif, PIF motif, and toggle switch were highlighted. **b** Structural comparison of active B2R with inactive M1R (yellow-orange, PDB ID: 5CXV), and inactive H_1_R (teal, PDB ID: 3RZE). DRY motif, NPxxY motif, PIF motif, and toggle switch were highlighted with the interactions labelled by dotted lines.

Superposition of our determined active B2R structures with the doxepin-bound inactive H_1_R^45^, histamine-bound active H_1_R^24^, tiotropium-bound inactive M1R^46^, and iperoxo-bound active M1R^33^ structures provided the molecular insights into the activation mechanisms of B2R. In the inactive H_1_R and M1R structures, R^3.50^ of the conserved DRY motif formed hydrogen bonds with the residues in TM6, and locked the receptor in an inactive state (Fig. 5b). Additionally, N^7.49^ of the conserved NPxxY motif formed hydrogen bonds with aspartic acids in TM2. While in the active structures, the DRY, NPxxY, and PIF motifs exhibited similar conformations, indicating the conserved activation mechanism across the class A GPCRs (Fig. 5a).

The B2R antagonist icatibant showed a different chemical structure from bradykinin and kallidin in the P4^K^/P3^B^, F6^K^/F5^B^, P8^K^/P7^B^, and F9^K^/F8^B^. Especially, F9^K^/F8^B^ in bradykinin and kallidin induced the side chain displacement and rotation of the conserved toggle switch W283^6.48^ (Supplementary Fig. 6c) thus influencing the P^5.50^I^3.40^F^6.44^ motif, which was found in many GPCRs as an allosteric bridge to coordinate the communications between the ligand-binding pockets and G protein-coupling interfaces^47^. It was suggested that the translocation of F^6.44^ in the PIF motif induced the intracellular side of TM6 extending outward from the TMs core. Upon TM6 displacement, hydrogen bonds within DRY-TM6/ICL2 and NPxxY-TM2 were destructed to allow these two motifs interacting with G proteins. In our determined B2R structure, the hydrogen bonds of D154^3.49^-Y356^G.H5.23^ and R155^3.50^-Y356^G.H5.23^ were found between DRY motif and α5-helix. Besides, the additional hydrogen bonds were observed between D154^3.49^ and R169^ICL2^ to stabilize the helical structure of ICL2, as well as between NPxxY motif and α5-helix (Y332^7.53^-N357^G.H5.24^) (Fig. 5b). Therefore, rearrangements of these important microswitches, as well as displacements of intracellular helices and loops, might facilitate B2R activation and G_q_ coupling. The C-terminal F8 of AngII was also important for AT_1_R and AT_2_R activation since it triggered a series of conformational changes^38,39^. The side chain of F8 in AngII formed a hydrogen bond with K^5.42^ of AT_1_R and AT_2_R, while the side chain of F9^K^ in kallidin orientated to the opposite direction with no hydrogen bond formed (Supplementary Fig. 13e). The bulky side chain of F8 in AngII pushed W253^6.48^ and Y292^7.43^ of AT_1_R downward to avoid steric clashes, and consequently promoted conformational changes at the toggle switch and PIF motif, which further induced TM6 outward displacement. Upon activation, the internal lock between N111^3.35^ and N295^7.46^ that stabilized the inactive AT_1_R was disrupted^29,39,40^. AT_2_R shared a similar activation process except for the absence of an internal lock between N127^3.35^ and S311^7.46^ of AT_2_R^29,38^. Therefore, the C-terminal phenylalanines of the peptides bradykinin and AngII triggered the B2R and AT_1_R/AT_2_R activation with conserved mechanisms.

## Discussion

Our determined B2R-G_q_ complex structures provided deep insights into the molecular mechanisms of the ligand binding, receptor activation, and G protein coupling of the human bradykinin receptor. The peptide agonists bradykinin and kallidin adopted the S-shaped binding poses. The N/C-termini of the peptides fell into the anion trap located at the entrance of the orthosteric ligand-binding pocket and were stabilized by the electrostatic interactions. To accommodate the hydrophobic residues in the middle of peptides, B2R formed hydrogen bonds with proline and glycine of the ligands. These two residues could act as the anchor points to immobilize the backbones of agonists. Divergences of the peptide-receptor interactions between B2R and B1R mainly focused on the C-termini of peptides. In B1R^37^, the carboxyl group of F9^DK^ was negatively charged and fell into a cationic pocket formed by K^3.33^ and R^5.38^. However, F8^B^ was electroneutral and only hydrophobic interactions could be found (Supplementary Fig. 12b). Additionally, the pocket consisting of R^5.38^, Y^6.51^, and E^6.58^ in B1R was infeasible to accommodate R9^B^, because of the steric hindrance with R^5.38^ (Supplementary Fig. 12c).

G_q_ formed substantial interactions with ICL2, ICL3, and helix 8 of B2R. ICL3 was usually truncated to increase the thermostability of GPCR-G protein complex for structure determination^48^, which made it difficult to investigate the roles of ICL3 in the G protein coupling. In our structures, ICL3 was found to grasp the α5-helix through polar interaction of N254^5.68^-Q350^G.H5.17^ and electrostatic force of K260^6.25^-D346^G.H5.13^ as well as hydrophobic interactions (Fig. 3b). Helix 8-G_q_ interactions were unusual in the previously determined GPCR-G_q/11_ structures^24,49^, which might further induce inward movement of TM7 at the cytoplasmic side during activation.

Our structures of B2R-G_q_ complexes also indicated the activation mechanisms of B2R. Upon bradykinin or kallidin binding to the bulky orthosteric binding pocket, F8^B^/F9^K^ interacted with the conserved toggle switch W283^6.48^, and then induced outward movement of TM6 at the cytoplasmic side and F279^6.44^ in the PIF motif. The rearrangement of PIF motif destabilized the inactive state of the DRY and NPxxY motifs, opened the intracellular cleft for the α5-helix insertion of G_q_. The active B2R-G_q_ complex structures we determined in this study are thus expected to pave the way to the structure-based drug design of B2R ligands for the treatment of cardiovascular diseases and COVID-19.

## Methods

### Constructs

To increase the yields and stability of the complex, the first 28 residues of human B2R were replaced by a maltose-binding protein (MBP) and the gene was cloned into pFastbac1 that contained a haemagglutinin (HA) tag, a FLAG tag (DYKDDDDK), a 10xHis tag, and a TEV protease cleavage site before the N-terminus of MBP. A DNGα_q_ was generated by introducing the mutations R183Q and Q209L, to decrease the binding affinity of GDP to G_q_, while the human Gβ_1_ and Gγ_2_ proteins were both wild types^22,23^. There was a 10xHis tag at the N-terminus of each subunit.

### Expression and purification of B2R-G_q_ complexes

The human B2R, DNGα_q_, Gβ_1_ and Gγ_2_ were co-expressed in sf9 insect cells. Cell cultures were grown to a density of 2 million cells per ml in ESF921 serum-free media (Expression Systems). Then the cells were infected by adding the baculoviruses of B2R, DNGα_q_-Ric8A and Gβ_1_γ_2_ at the ratio of 1:1:1. After 48 h incubation at 27 °C, shaking at 125 rpm, cells were harvested by centrifugation and stored at −80 °C until use.

Cell pellets infected with B2R and G_q_ heterotrimer were thawed at room temperature and suspended by dounce homogenization in 20 mM HEPES pH 7.5, 50 mM NaCl, 2 mM MgCl_2_, 500 μM AEBSF, 1 μM E-64, 1 μM Leupeptin, 150 nM Aprotinin. The complex was formed on membranes in the presence of 10 μM bradykinin (Sangon Biotech) or 5 μM kallidin (Alomone), and was treated with apyrase (100 mU/ml, NEB) and β-mercaptoethanol (β-ME, 2 mM), followed by 3 h incubation at room temperature. Membranes were collected by centrifugation at 30,000 × *g* for 30 min. The washed membranes were resuspended and solubilized in 60 mM HEPES pH 7.5, 100 mM NaCl, 0.5% lauryl maltose neopentyl glycol (LMNG, Anatrace), 0.05% cholesteryl hemisuccinate Tris salt (CHS, Anatrace), 10 mM imidazole, 2 mM MgCl_2_, 50 mU/ml apyrase, 1 mM β-ME supplemented with 5 μM bradykinin or 2.5 μM kallidin. The membranes were incubated at 4 °C for 4 h. Insoluble material was removed by centrifugation at 58,000 × g for 1 h and the supernatant was incubated with pre-equilibrated Talon IMAC resin overnight. Then the resin was packed into a gravity column (Bio-Rad) and washed with 15 column volumes of 50 mM HEPES pH 7.5, 100 mM NaCl, 0.05% LMNG, 0.005% CHS, 20 mM imidazole, 2 mM MgCl_2_, 5 μM for bradykinin or 2.5 μM for kallidin, and 15 column volumes of 20 mM HEPES pH 7.5, 100 mM NaCl, 0.002% LMNG, 0.0002% CHS, 40 mM imidazole, 2 mM MgCl_2_, 5 μM for bradykinin or 2.5 μM for kallidin. The protein was eluted in 5 column volumes of 20 mM HEPES pH 7.5, 100 mM NaCl, 0.002% LMNG, 0.0002% CHS, 300 mM imidazole, 2 mM MgCl_2_, 5 μM for bradykinin or 2.5 μM for kallidin. The eluted material was incubated with 100 mU/ml apyrase and 2 mM β-ME for 2 h at 4 °C followed by incubation with pre-equilibrated amylose resin (NEB) for 4 h at 4 °C. The resin was loaded onto a gravity column and washed with 10 column volumes of 20 mM HEPES pH 7.5, 100 mM NaCl, 0.002% LMNG, 0.0002% CHS, 2 mM MgCl_2_, 5 μM bradykinin or kallidin. The protein was eluted in 6 column volumes of 20 mM HEPES pH 7.5, 100 mM NaCl, 0.002% LMNG, 0.0002% CHS, 2 mM MgCl_2_, 5 μM bradykinin or kallidin, and 10 mM maltose. Eluted protein was concentrated using a Vivaspin Turbo Ultrafiltration Units (MWCO 50 kDa) and subjected to size-exclusion chromatography on a Superdex 200 Increase 10/300 column (GE Healthcare) pre-equilibrated with 20 mM HEPES pH 7.5, 100 mM NaCl, 0.002% LMNG, 0.0002% CHS, 2 mM MgCl_2_, 0.5 μM bradykinin or kallidin. Peak fractions containing B2R-G_q_ complex were pooled and concentrated to ~4 mg/ml for electron microscopy studies. The final yields of the purified complexes were ~0.15 mg/L insect cell culture.

### Cryo-EM grid preparation and data collection

For cryo-EM grids preparation, 3 μL B2R-G_q_ complex solution was applied to glow-discharged holey carbon EM grids (C-flat 300 Cu mesh R1.2/1.3) using Vitrobot (FEI Vitrobot Mark IV). The grid was plunge-freezing into liquid ethane and stored in liquid nitrogen for further data collection.

Cryo-EM data collection was performed on a Titan Krios (ThermoFisher) electron microscope operated at 300 kV equipped with a K2 Summit direct electron detector (Gatan) at a magnification of 29,000× in the Center of Cryo-Electron Microscopy, Zhejiang University (Hangzhou, China), corresponding to a nominal pixel size of 1.014 Å. Movies were recorded using SerialEM software in counting mode at a dose rate of 8.0 e/Å^2^/s with a defocus range of −0.5 to −2.0 μm. The total exposure time was 8 s and a total of 40 frames per micrograph. A total of 4093 and 3425 movies were collected for B2R-G_q_-bradykinin and B2R-G_q_-kallidin, respectively.

### Cryo-EM data processing and structure determination

The flow charts of data processing were presented in Supplementary Figs. 3 and 4. For three batches of movie stacks, global motion correction was performed using the MotionCorr2 program^50^, and contrast transfer function (CTF) parameters were estimated using CTFFIND4^51^. The remaining image processing steps were carried out using RELION 3.0^52^.

For the B2R-G_q_-bradykinin complex, 2,630,394 particles were picked from 4,093 movies using the Laplacian-of-Gaussian-based auto-picking method and binned three times before 2D classification. The iterative 2D classification caused 1,169,656 well-qualified particles, which were then selected for further 3D classification. Using TT-OAD2–GLP-1R–G_s_ complex (EMDB-20179)^53^ as a reference, the 3D classification resulted in 1,056,238 well-defined particles, which were then re-extracted using the original pixel size of 1.014 Å and used for 3D refinement, CTF refinement, and Bayesian polishing. The final refinement generated a map with an indicated global resolution of 2.93 Å at a Fourier shell correlation of 0.143.

For the B2R-G_q_-kallidin complex, the Laplacian-of-Gaussian-based auto-picking produced 2,042,659 particles, which were binned 3 times and subjected to integrative 2D classifications. Particles from qualified 2D averages were then selected for further 3D analysis using the map of B2R-G_q_-bradykinin complex as the reference, resulting in a well-defined subset with 1,388,486 particles. The particles were re-extracted using the original pixel size of 1.014 Å and subjected to 3D refinement, particle polishing and CTF refinement. The final refinement yielded a map with a resolution of 2.76 Å at FSC = 0.143.

Cryo-EM structure models were built using the cryo-EM structure of M1R-G_11_ (PDB ID: 6OIJ)^33^ as the initial model. The model was docked into the EM density maps using Chimera and manually rebuilt using COOT^54^. Realspace refinement was performed using Phenix^55^. The final refinement statistics were validated using Phenix and shown in Supplementary Table 2. All the figures were prepared using PyMol^56^ and UCSF Chimera^57^.

### Calcium mobilization assays

CHO cells transfected with wild-type or mutated B2R and wild-type or mutated G_q_ were seeded into 96-well black plates at a density of 30,000 cells per well and incubated for 24 h. Then cells were loaded with reagents from Calcium-5 Assay Kit (Molecular Devices) for 45 min at 37 °C in 5% CO_2_ according to the manufacturer’s protocol. Cells were treated with varying concentrations of kallidin and detected with Flexstation 3 Multi-Mode Microplate Reader (Molecular Devices) with excitation at 485 nm and emission at 525 nm. Data were analyzed by GraphPad Prism 5 and presented as Mean ± S.E.M. from three independent experiments.

### Reporting summary

Further information on research design is available in the Nature Research Reporting Summary linked to this article.

## Supplementary information

Supplementary Information

Reporting Summary

## Data Availability

Cryo-EM maps have been deposited in the Electron Microscopy Data Bank under accession codes: EMD-31480 (B2R-G_q-_bradykinin) and EMD-31481 (B2R-G_q_-kallidin). The atomic coordinates have been deposited in the Protein Data Bank under accession codes: 7F6H (B2R-G_q_-bradykinin) and 7F6I (B2R-G_q_-kallidin). Source data are provided with this paper.

## Source data

Source Data
