# Supplementary material for: Cryo-EM structures of human bradykinin receptor-Gq proteins complexes
Source: Nat Commun. 2022 Feb 7;13:714. doi: 10.1038/s41467-022-28399-1 (PMC8821558; doi:10.1038/s41467-022-28399-1)
Supplement: Supplementary file 1 — Supplementary Information [file 41467_2022_28399_MOESM1_ESM.pdf]

Supplementary Information

Cryo-EM structures of human bradykinin receptor-G_q proteins complexes

Jinkang Shen[1], Dongqi Zhang[1], Yao Fu[1], Anqi Chen[1], Xiaoli Yang[1], Haitao Zhang[1,2]

[1]Hangzhou Institute of Innovative Medicine, Institute of Pharmacology and Toxicology, Zhejiang Province Key Laboratory of Anti-Cancer Drug Research, College of Pharmaceutical Sciences, Zhejiang University, Hangzhou 310058, Zhejiang, China

[2]The Second Affiliated Hospital, Zhejiang University School of Medicine, Hangzhou 310009, Zhejiang, China.

Correspondence: Haitao Zhang (haitaozhang@zju.edu.cn)

Supplementary Figures 1-13

Supplementary Tables 1-3

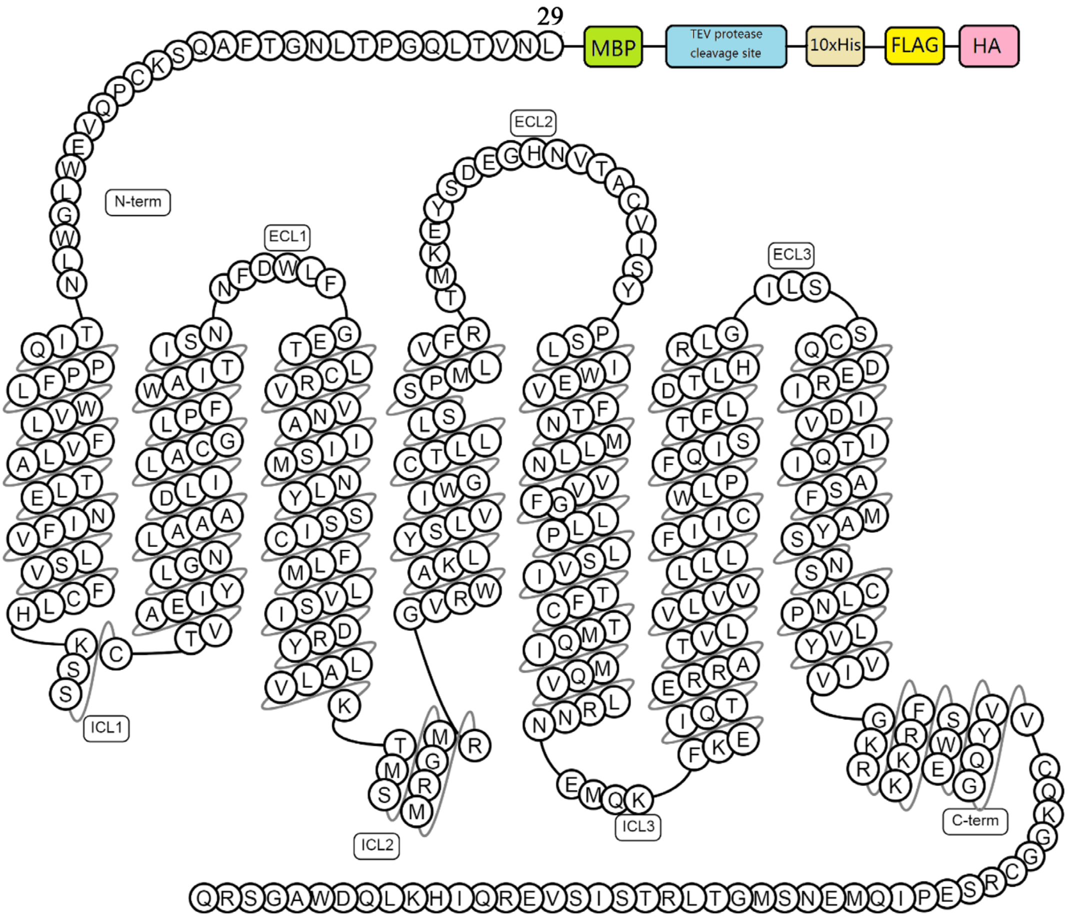

Supplementary Figure 1. Snake plot of B2R construct design. The N-terminal 28 residues were truncated and the HA-Flag-His_{10}-TEV-MBP tags were added at the N-terminus.

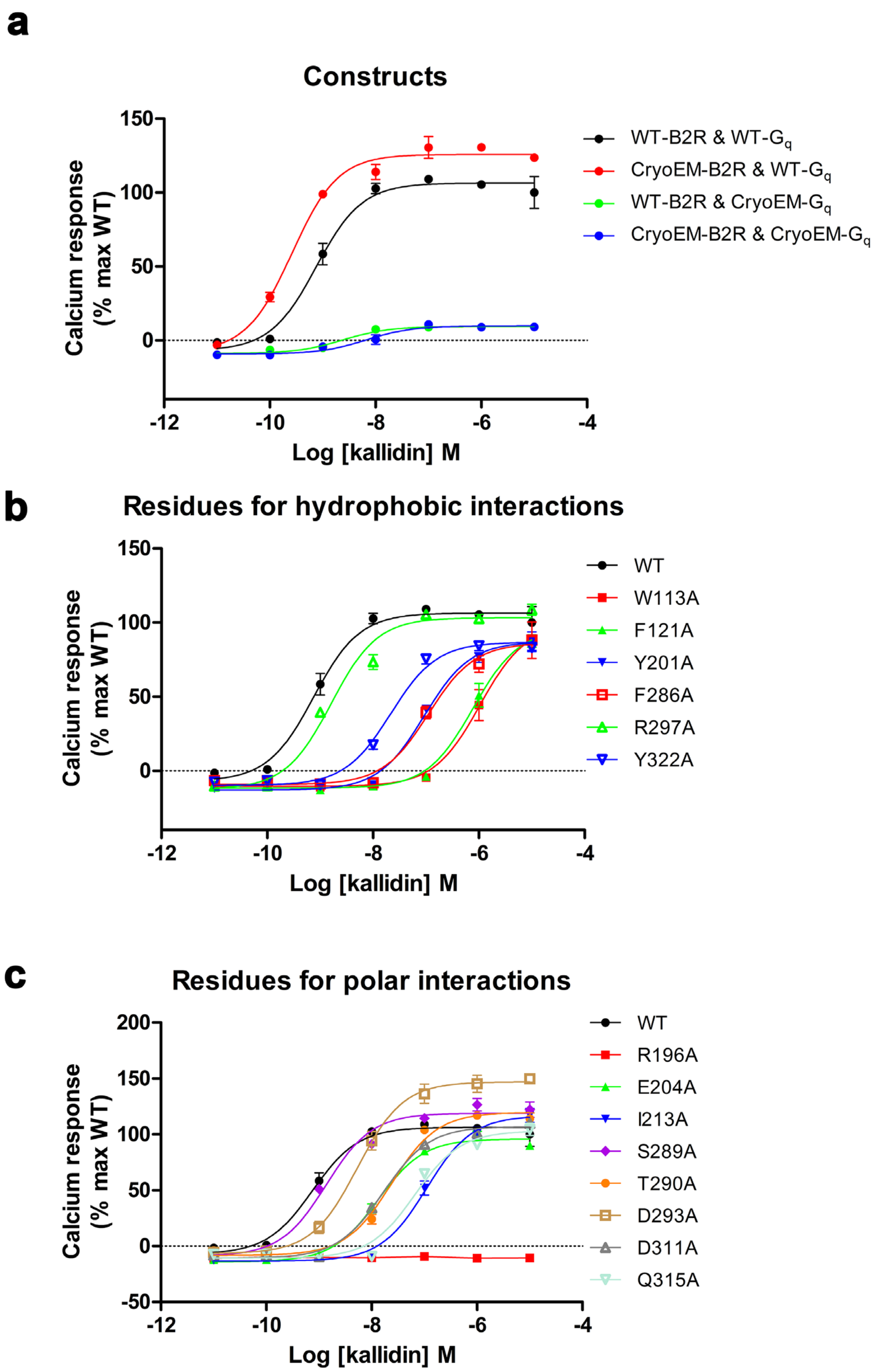

Supplementary Figure 2. Kallidin dose-dependent calcium mobilization results of WT and mutated B2R. a-c Comparison of WT-B2R and CryoEM-B2R (**a**), alanine mutations of residues for hydrophobic interactions (**b**), and polar interactions (**c**) on kallidin-induced B2R activation. Data represented the Mean ± S.E.M., *n*=3 independent replicates. WT represented wild type. Source data are provided as a Source Data file.

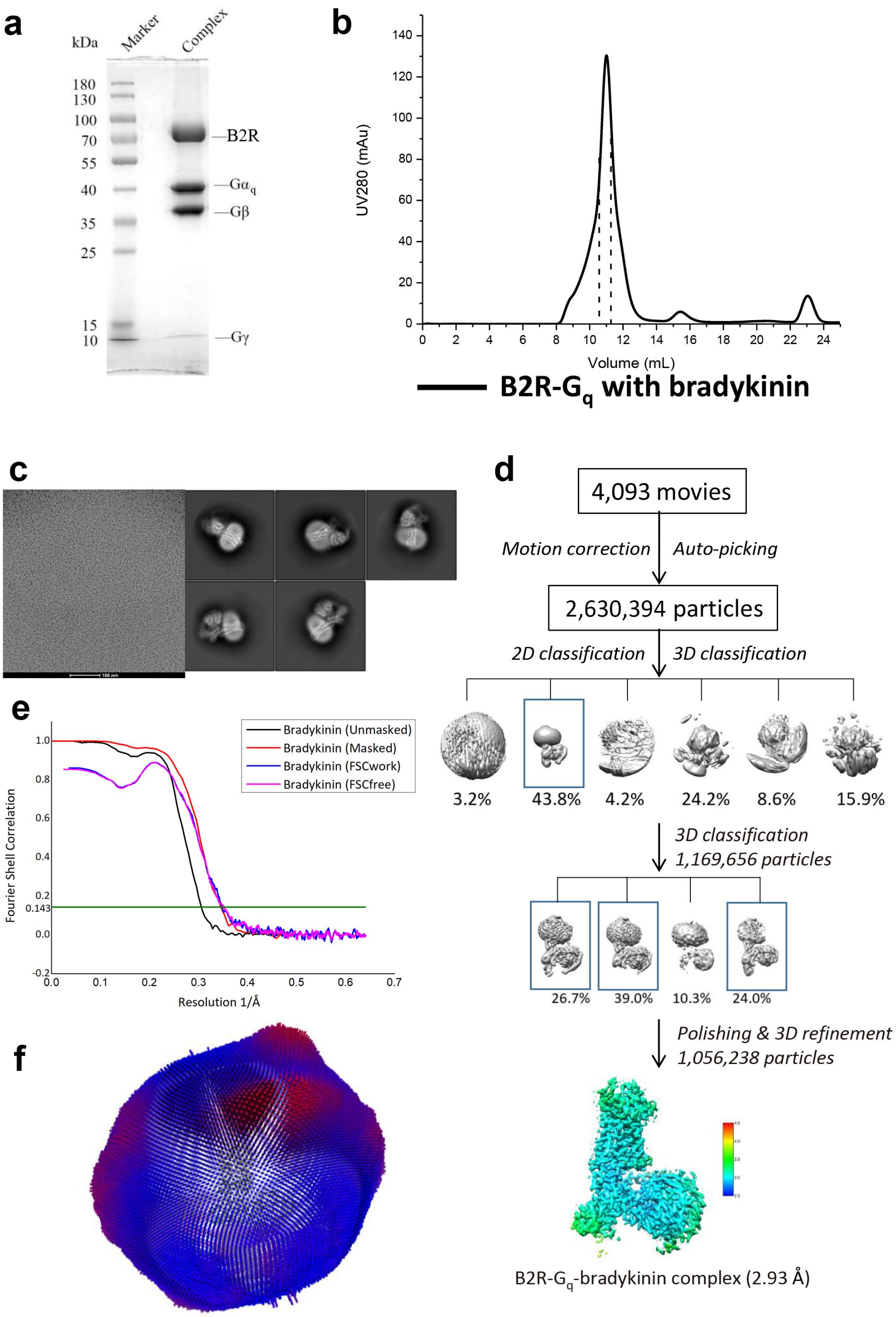

Supplementary Figure 3. Purification and cryo-EM data analysis of B2R-G_q-bradykinin complex. a SDS-PAGE analysis of purified B2R-G_q-bradykinin complex. **b** Representative size-exclusion chromatography elution profile of the purified B2R-G_q-bradykinin complex using Superdex 200 Increase 10/300 column (GE Healthcare). **c-f** Cryo-EM structure determination with micrograph/2D classification (**c**), data processing workflow (**d**), resolution (**e**) and angle distribution map (**f**). Each experiment was repeated twice independently with similar results. Source data are provided as a Source Data file.

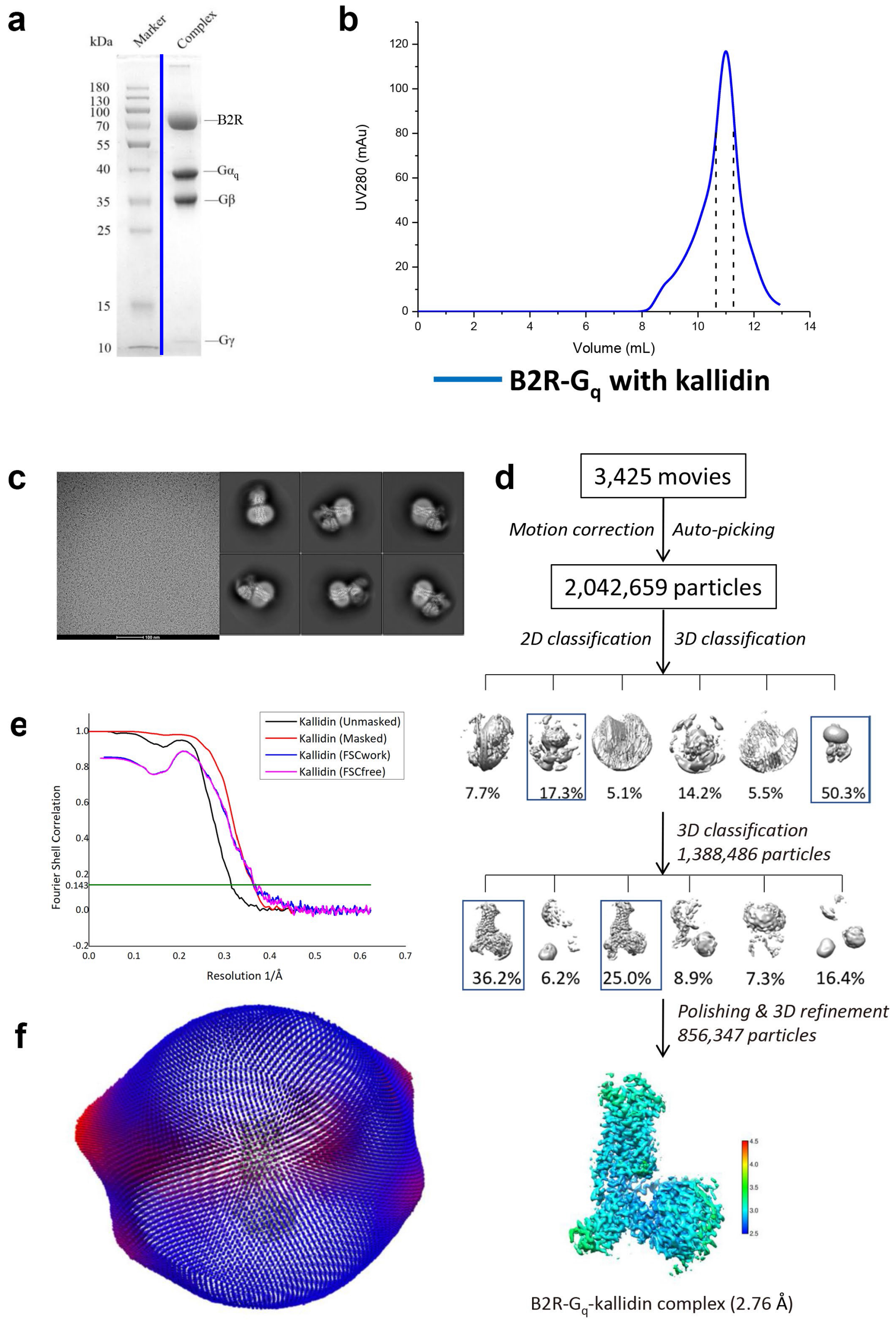

Supplementary Figure 4. Purification and cryo-EM data analysis of B2R-G$_q$-kallidin complex. **a** SDS-PAGE analysis of purified B2R-G$_q$-kallidin complex. **b** Representative size-exclusion chromatography elution profile of the purified B2R-G$_q$-kallidin complex using Superdex 200 Increase 10/300 column (GE Healthcare). **c-f** Cryo-EM structure determination with micrograph/2D classification (**c**), data processing workflow (**d**), resolution (**e**) and angle distribution map (**f**). Each experiment was repeated twice independently with similar results. Source data are provided as a Source Data file.

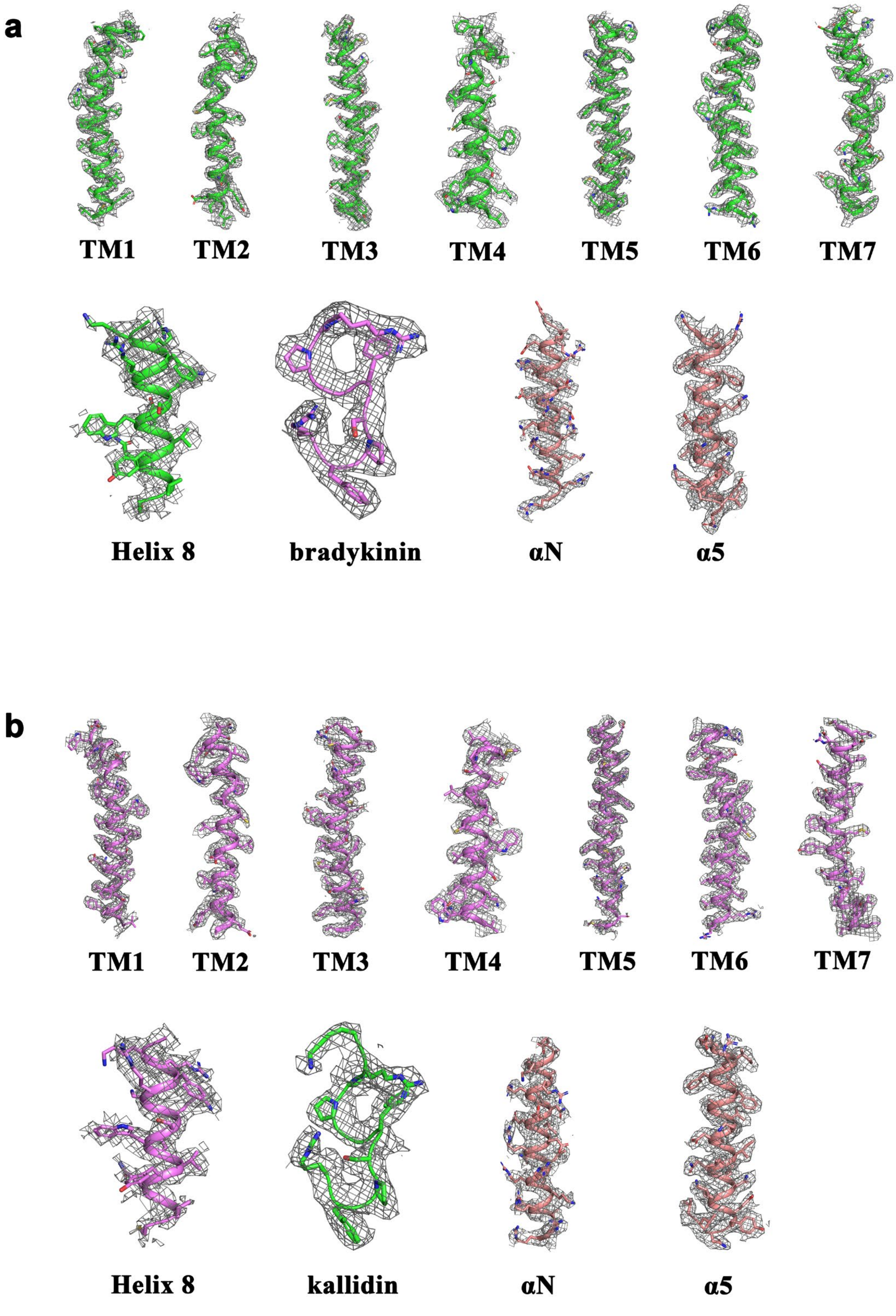

Supplementary Figure 5. Cryo-EM density maps. a, b Representative cryo-EM density maps of transmembrane helices and helix 8 of B2R, αN-helix and α5-helix of G_q protein, the peptide agonists bradykinin and kallidin in the B2R-G_q-bradykinin (**a**) and B2R-G_q-kallidin (**b**) structures, respectively. Cryo-EM density maps were set at contour level of 5.0.

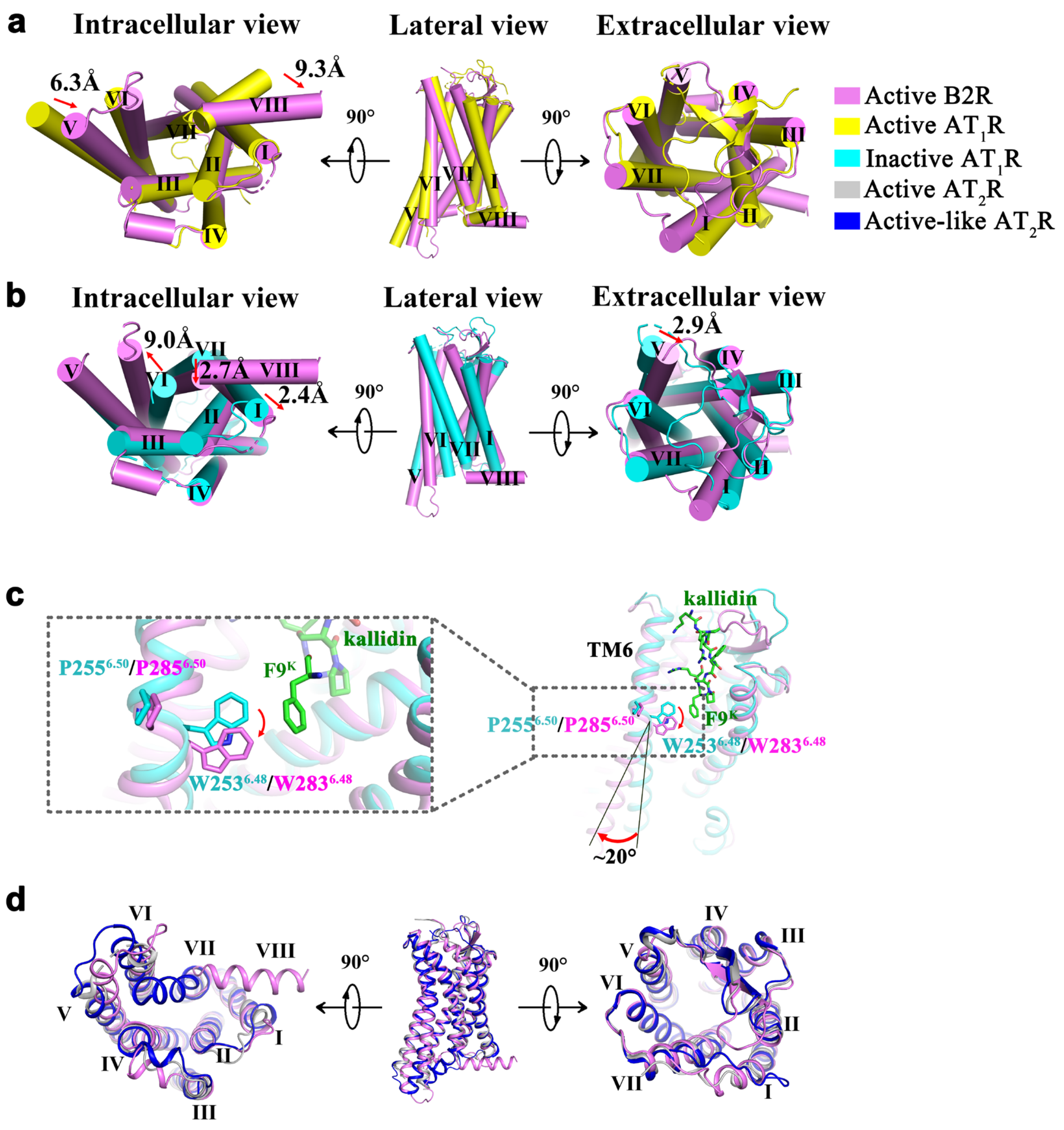

Supplementary Figure 6. Structural divergences among B2R, AT_1R and AT_2R. a Structural comparison of active B2R (violet) and active AT_1R (PDB ID: 6DO1, yellow). Red arrows represented the conformational differences of TM5 and helix 8. **b** Structural comparison of active B2R (violet) and inactive AT_1R (PDB ID: 4ZUD, cyan). Red arrows represented the conformational differences of TM1, TM5, TM6, and TM7. **c** Lateral view of the interactions between toggle switch ($W283^{6.48}$) and $F9^K$ in active B2R structure. Intracellular side of TM6 underwent a ~20° outward rotation upon agonist binding. **d** Structural comparison of active B2R (violet), agonist-bound active AT_2R (PDB ID: 5XJM, grey) and antagonist-bound active-like AT_2R (PDB ID: 5UNG, blue).

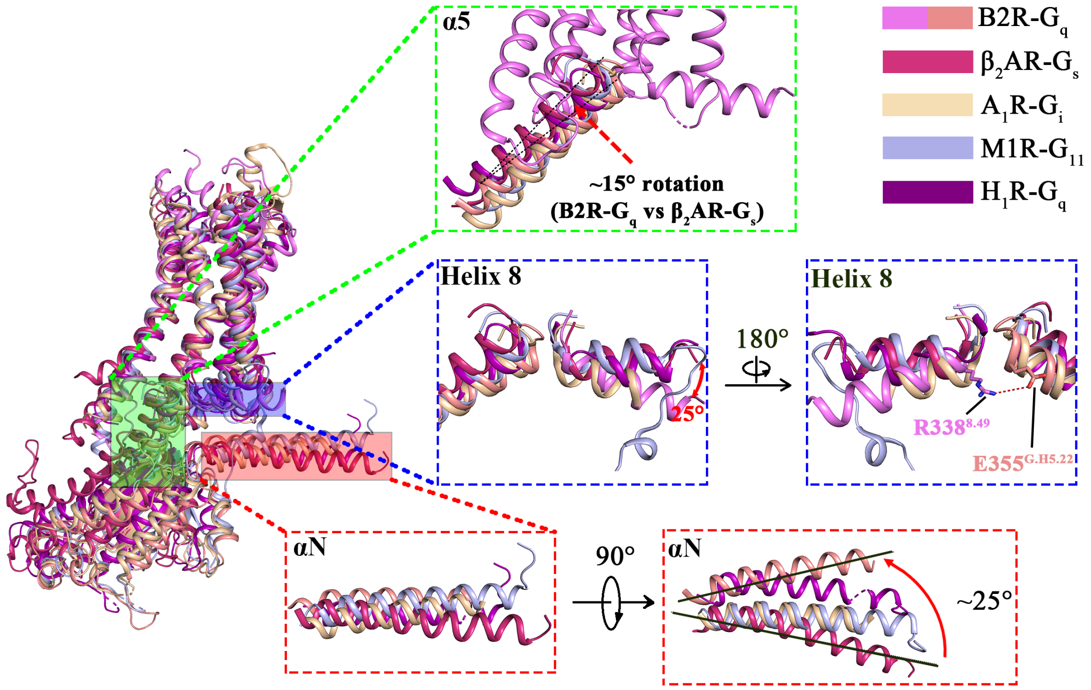

Supplementary Figure 7. Comparison with other GPCR-G protein complexes. Superposition of B2R-G_q (B2R, violet; G_q, salmon), β_2AR-G_s (warm-pink), A_1R-G_i (wheat), M1R-G_{11} (light-blue), and H_1R-G_q (purple), with helix 8, α5-helix, and αN-helix highlighted.

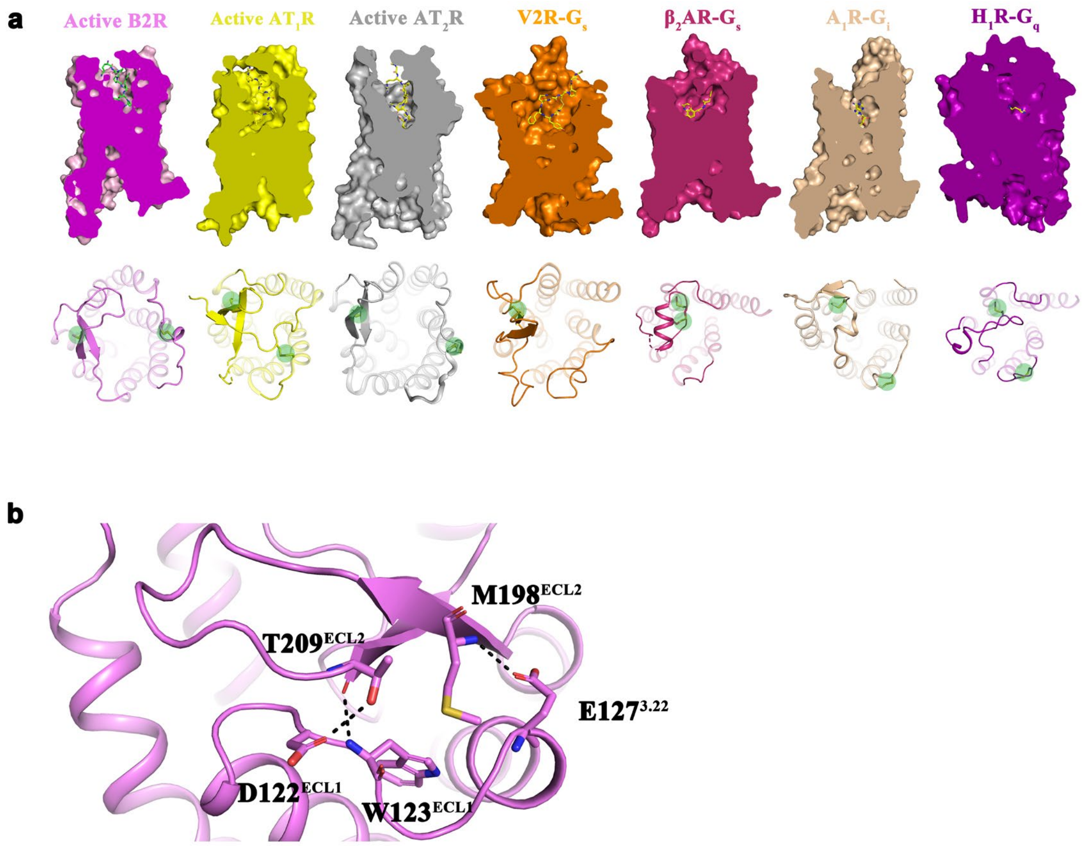

Supplementary Figure 8. Comparison of the orthosteric ligand-binding pockets of B2R with other class A GPCRs. a Surfaces sliced to expose the binding pockets and the corresponding top views of ECLs were shown for kallidin-bound B2R (B2R, violet; kallidin, green), s-AngII-bound AT_1R (yellow, PDB ID: 6DO1), s-AngII-bound AT_2R (grey, PDB ID: 5XJM), arginine-vasopressin-bound V2R (orange, PDB ID: 7BB6), BI-167107-bound β_2AR (warm-pink, PDB ID: 3SN6), adenosine-bound A_1R (wheat, PDB ID: 6D9H), and histamine-bound H_1R (purple, PDB ID: 7DFL). Disulfide bonds formed within ECLs were highlighted by green circles. **b** Intramolecular hydrogen bonds between ECL1 and ECL2 in B2R-G_q structure were shown as dashed lines.

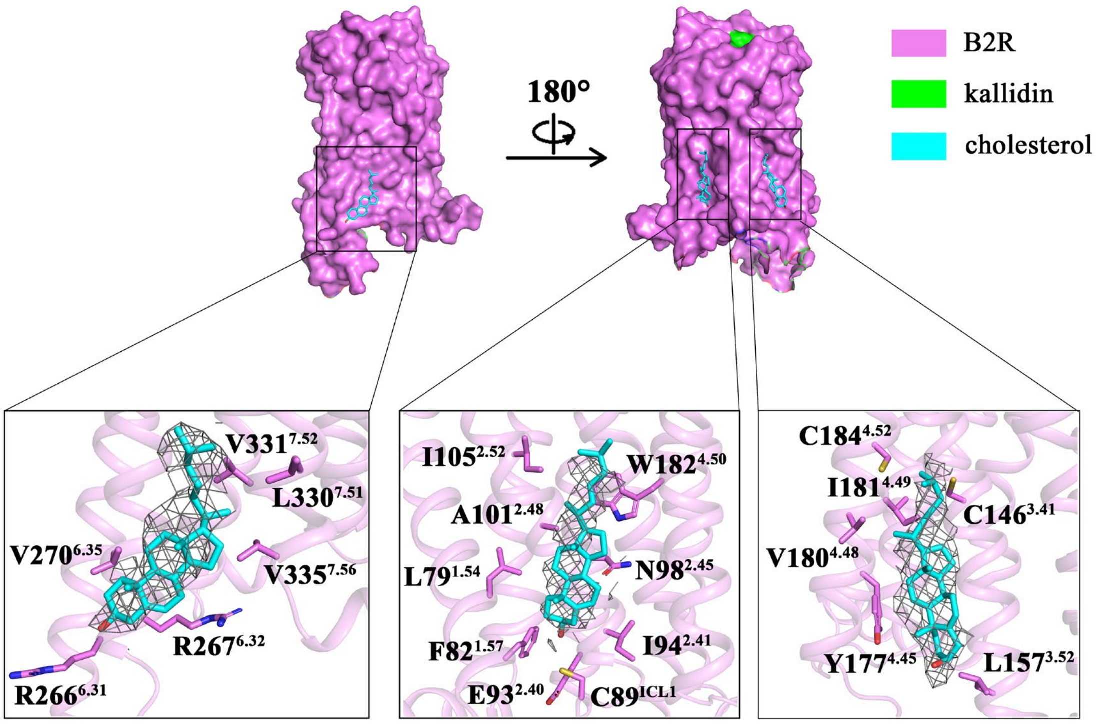

Supplementary Figure 9. Interactions between B2R and cholesterols. Three structured cholesterols (cyan) lay in intracellular clefts between TM2-TM4, TM3-TM4, and TM6-TM7. Residues locating around cholesterols were shown as violet sticks. The density maps of cholesterols were set at contour level of 5.0.

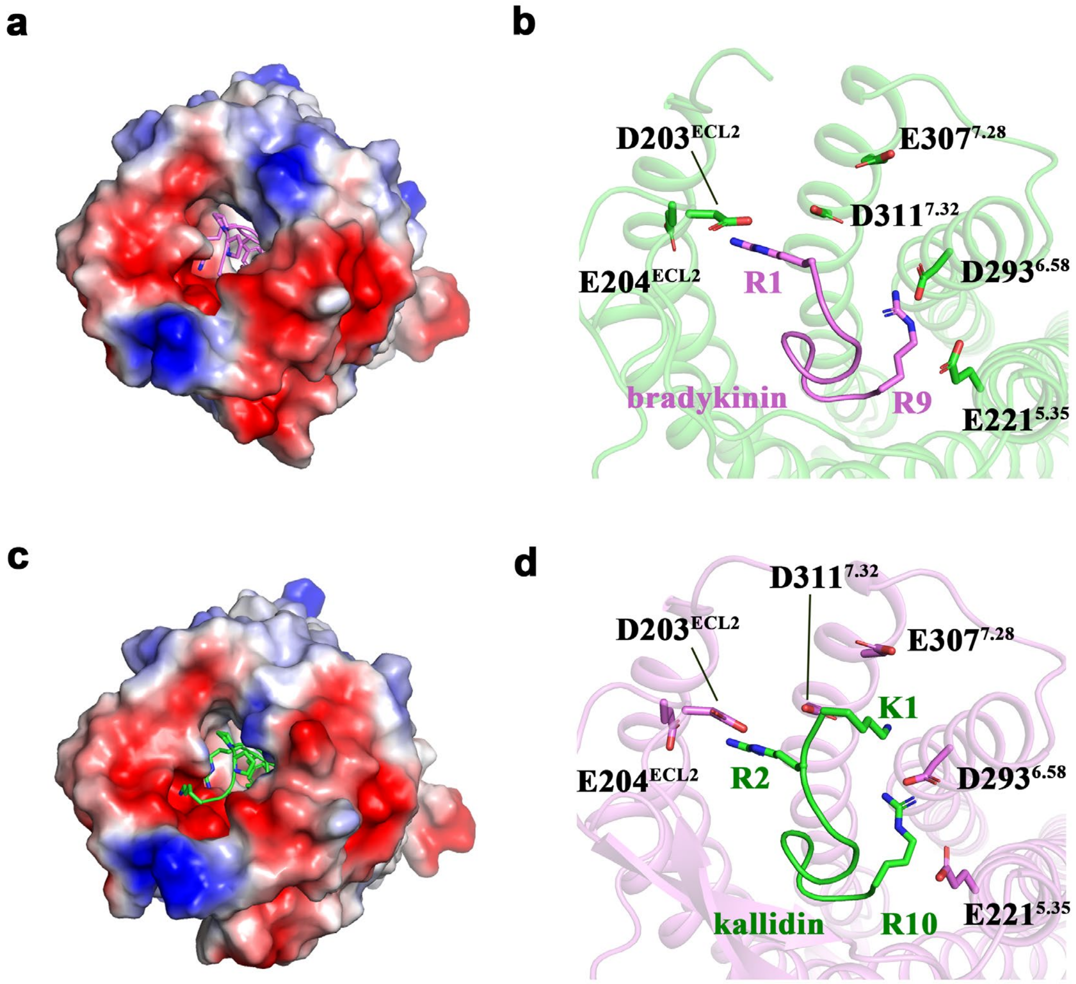

Supplementary Figure 10. Vacuum electrostatic surfaces in the B2R-G_q structures. **a** Electrostatic surface of bradykinin-bound B2R with bradykinin shown as violet sticks. **b** Anion trap was formed by aspartic acids and glutamates locating at the entrance of the binding pocket (B2R, green; bradykinin, violet). **c** Electrostatic surface of kallidin-bound B2R with kallidin shown as green sticks. **d** Anion trap was formed by aspartic acids and glutamates locating at the entrance of the binding pocket (B2R, violet; kallidin, green).

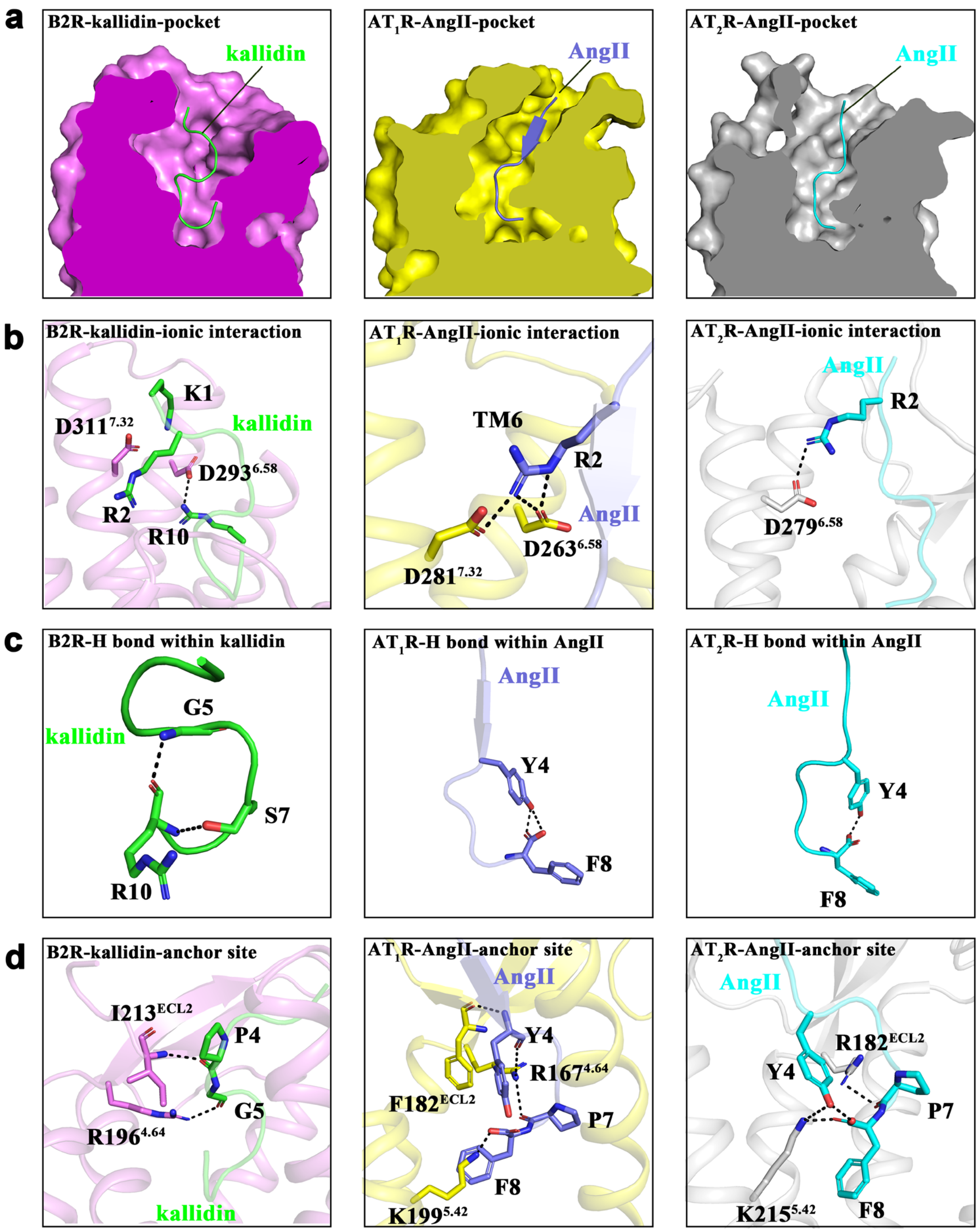

Supplementary Figure 11. Comparison of ligand-binding among B2R, AT_1R and AT_2R. a Overall architectures of the orthosteric ligand-binding pockets of B2R, AT_1R (PDB ID: 6OS0), and AT_2R (PDB ID: 6JOD), with kallidin and AngII. **b** $D^{6.58}$ and $D^{7.32}$ located at the entrance of the pockets and were highly conserved across these receptors. Dotted lines indicated the ionic interactions between agonists and aspartic acids. **c** Intramolecular hydrogen bonds of kallidin and AngII. **d** Hydrophobic residues of agonists anchored the ligands in the pockets.

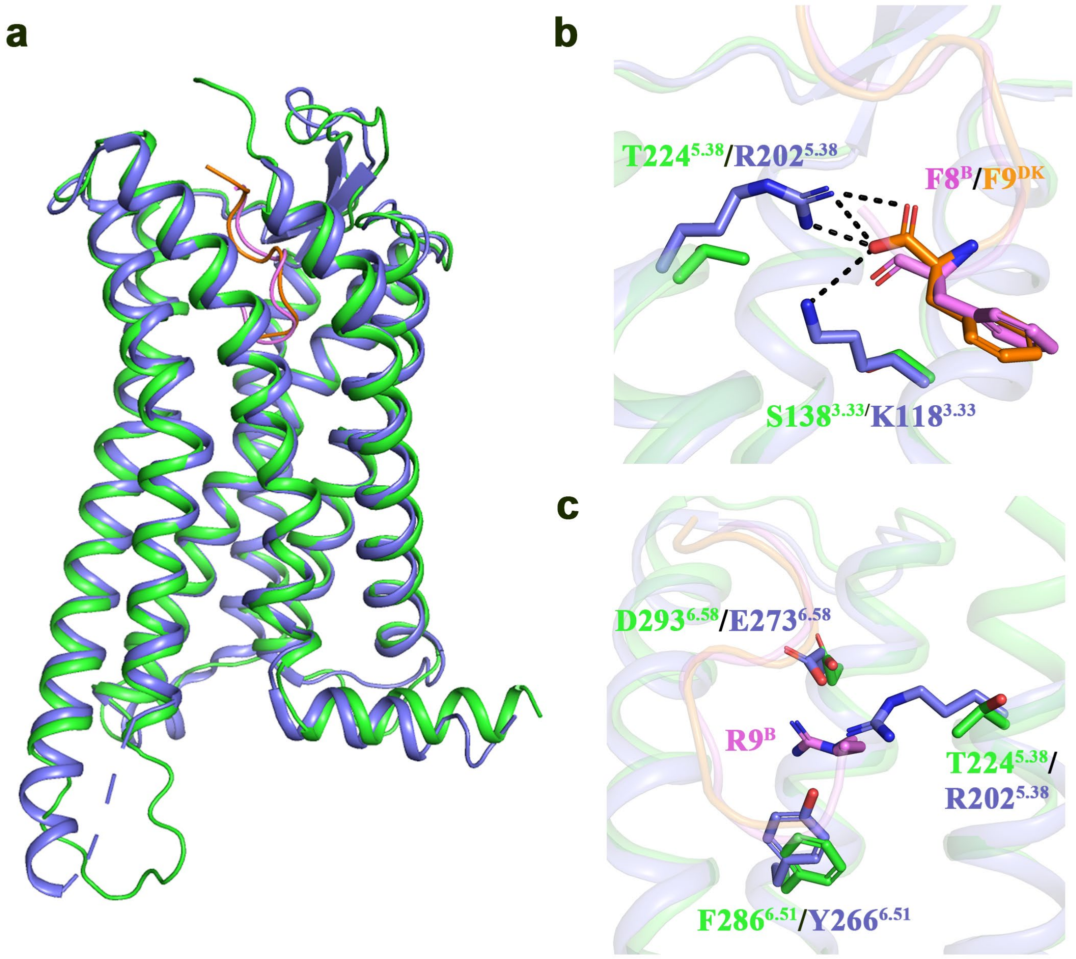

Supplementary Figure 12. Structural comparison between active B1R and B2R. **a** Comparison of bradykinin-bound B2R and desArg^{10}-kallidin bound B1R (PDB ID: 7EIB, desArg^{10}-kallidin, orange; bradykinin, violet; B1R, slate; B2R, green). **b, c** Key residues responsible for ligands selectivity between B1R and B2R.

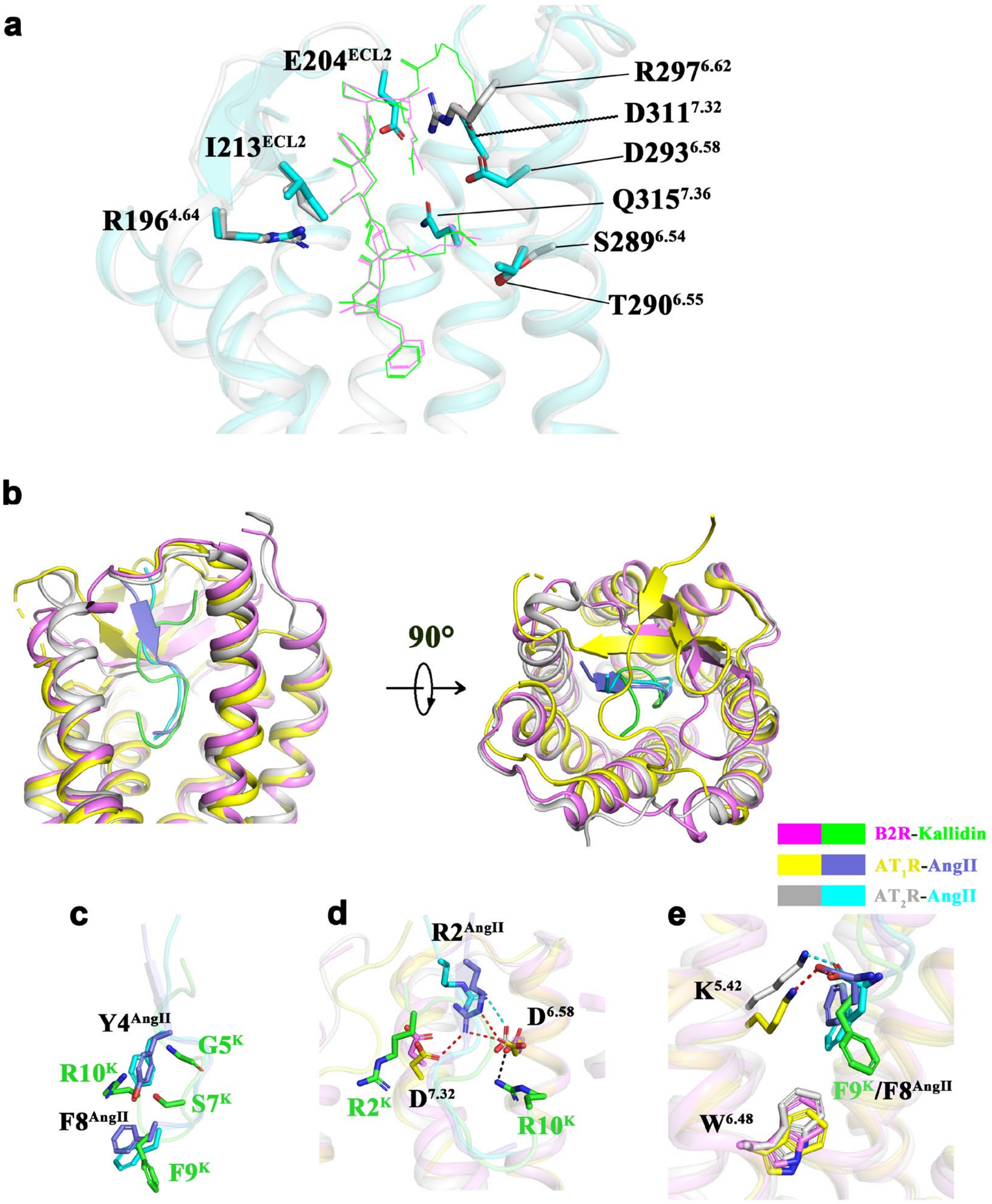

Supplementary Figure 13. Comparison of bradykinin-bound and kallidin-bound B2R, and angiotensin receptors. a Superposition of bradykinin and kallidin as well as key residues in the ligand-binding pockets (bradykinin, violet; kallidin, green; bradykinin-bound B2R, grey; kallidin-bound B2R, cyan). **b** Ligand-binding pockets of kallidin-bound B2R, AngII-bound AT_1R (PDB ID: 6OS0) and AT_2R (PDB ID: 6JOD). **c** Conformational comparison of kallidin and AngII in AT_1R and AT_2R. Residues for intramolecular hydrogen bonds were highlighted. **d, e** Residues involved in the ligand-binding (**d**) and receptor activation (**e**). Hydrogen bonds between B2R-kallidin (black), AT_1R-AngII (red), and AT_2R-AngII (cyan) were presented as dashed lines.

Supplementary Table 1. Calcium mobilization results of WT and mutated constructs. E_{max} and pEC_{50} values were presented as Mean±S.E.M. from three independent experiments. Source data are provided as a Source Data file.

**B2R**	**G_q**	**E_{max}**	**pEC_{50}**
**WT**	**WT**	106.4 ± 2.797	9.123 ± 0.095
**CryoEM**	**WT**	125.9 ± 2.185	9.581 ± 0.077
**WT**	**CryoEM**	9.342 ± 0.900	8.622 ± 0.181
**CryoEM**	**CryoEM**	9.746 ± 1.265	8.176 ± 0.213

Supplementary Table 2. Cryo-EM data collection, refinement, and validation statistics.

	**B2R-G_q-bradykinin**	**B2R-G_q-kallidin**
**PDB ID/EMDB ID**	**7F6H/EMD-31480**	**7F6I/EMD-31481**
**Data collection and processing**		
Magnification	29,000	29,000
Voltage (kV)	300	300
Electron exposure (e-/Å2)	64	64
Defocus range (μm)	-0.5 ~ -2.0	-0.5 ~ -2.0
Pixel size (Å)	1.014	1.014
Symmetry imposed	C1	C1
Initial particle projections (no.)	2,630,394	2,042,659
Final particle projections (no.)	1,056,238	856,347
Map resolution (Å)	2.93	2.76
FSC threshold	0.143	0.143
Map resolution range (Å)	2.5-4.5	2.5-4.5
**Refinement**		
Initial model used	6OIJ	6OIJ
Model resolution (Å)	3.0	2.9
FSC threshold	0.5	0.5
Map sharpening *B* factor (Å^2)	-117.25	-99.76
Model composition		
Non-hydrogen atoms	7,342	7,361
Protein residues	917	915
B factors (Å^2)	57.71	41.44
R.m.s.deviations		
Bond lengths (Å)	0.010	0.007
Bond angles (°)	0.961	0.886
Validation		
MolProbity score	1.75	1.60
Clashscore	6.26	6.36
Rotamer outliers (%)	0.87	0.50
Ramachandran plot		
Favored (%)	96.24	96.23
Allowed (%)	3.76	3.77
Disallowed (%)	0	0

Supplementary Table 3. B2R-G_q mediated calcium mobilization results. E_{max} and pEC_{50} values were presented as Mean±S.E.M. from three independent experiments. NA represented not available. Source data are provided as a Source Data file.

**B2R**	$\boldsymbol{E_{max}}$	$\mathbf{pEC_{50}}$
**WT**	106.4 ± 2.797	9.123 ± 0.095
**W113A**	98.92 ± 5.455	5.980 ± 0.085
**F121A**	95.84 ± 4.638	6.111 ± 0.078
**R196A**	NA	NA
**Y201A**	87.11 ± 2.596	7.032 ± 0.063
**E204A**	95.98 ± 2.030	7.886 ± 0.056
**I213A**	116.8 ± 3.558	6.958 ± 0.066
**F286A**	86.19 ± 4.524	6.951 ± 0.114
**S289A**	118.9 ± 2.829	8.861 ± 0.082
**T290A**	119.8 ± 2.960	7.625 ± 0.069
**D293A**	147.0 ± 3.360	8.269 ± 0.073
**R297A**	103.2 ± 2.719	8.790 ± 0.087
**D311A**	107.1 ± 1.708	7.778 ± 0.044
**Q315A**	103.2 ± 3.619	7.176 ± 0.082
**Y322A**	86.76 ± 2.154	7.667 ± 0.067
